# Evaluation of molecular diagnosis in fungal keratitis. Ten years of experience

**DOI:** 10.1007/s12348-011-0019-9

**Published:** 2011-02-23

**Authors:** Consuelo Ferrer, Jorge L. Alió

**Affiliations:** 1 Molecular Biology, Research and Development Department, Vissum Corporación–Instituto Oftalmológico de Alicante, Avenida de Denia s/n, 03016 Alicante, Spain; 2 Division of Ophthalmology, Universidad Miguel Hernández, Alicante, Spain

**Keywords:** Diagnosis, Keratitis, Molecular diagnosis, Infection, Fungi

## Abstract

**Purpose:**

The aims of this study were to assess the utility of polymerase chain reaction (PCR) in diagnosing fungal keratitis in the last decade in our center and to review the molecular diagnosis of mycotic keratitis.

**Methods:**

A retrospective nonrandomized investigation was undertaken at Vissum Corporación Instituto Oftalmologico de Alicante to evaluate 27 corneal samples of 20 patients with proven fungal keratitis from January 2000 to December 2009. Corneal samples (21 corneal scrapings, 5 biopsies, and 1 cornea) were evaluated by Gram stain or calcofluor stain, culture, and PCR. The detection and molecular identification were carried out by DNA amplification and sequencing of the internal transcribed spacer and 5.8S rRNA region from the corneal samples.

**Results:**

PCR detected all the samples that were positive by conventional methods. Four samples were positive by PCR and showed negative results by culture and stain. Combination of microscopy and culture gave positive results in 21 of the 27 samples of patients with mycotic keratitis. Stains showed a 66.7% of positive results, culture showed 59.3%, and PCR showed 92.6%. The time taken for PCR assay was 4 to 8 h whereas positive fungal cultures took 1 to 35 days. Identification at species level by molecular methods was possible in all cases except one. Identification at species level by conventional methods only was possible in eight cases.

**Conclusions:**

PCR not only proved to be an effective rapid method for the diagnosis of fungal keratitis but was also more sensitive than stain and culture methods. Fungal PCR must be added as the screening diagnosis test when an early mycotic keratitis is suspected. Molecular identification is the gold standard technique for the identification of corneal fungal pathogens.

## Introduction

Microbial keratitis is a serious ocular infection that can cause corneal scarring and opacification. Approximately 28% of ulcerative keratitis shows mycotic origin, although these data vary from 6% to 53%, depending upon the country and especially on the climate [1–5]. The basis for an effective treatment is rapid diagnosis of the disease by the detection and identification of the causative agent. Approximately one decade ago, the diagnosis of fungal keratitis was based on culture and stain of the corneal scrapings. However, from this date, new diagnostic tools have been incorporated. The capacity for detection and identification of genomic material in any type of sample has allowed the diagnosis of many genetic or infectious diseases based on the DNA sequence. Molecular diagnosis of ocular infections is based on DNA detection of microorganisms by polymerase chain reaction (PCR) in ocular samples. The first report about the detection of fungal DNA in ocular samples was dated in 1996 by Alexandrakis et al. [6], but it was not until 1998 when Lohmann et al. [7] published the first work regarding the detection of fungal DNA based in universal primers. Since then, numerous laboratories have appeared around the word working on the DNA detection of ocular pathogens to carry out molecular diagnosis. Today, molecular diagnosis is carried out in the majority of ophthalmic clinics (directly or indirectly, sending the samples to reference laboratories). During this time, the techniques have changed to meet the needs of both ophthalmologists and patients, and the techniques are also easier, faster, and reproducible for the molecular biologist. In this work, we present our experience in this field in the last decade and review the literature about molecular diagnosis of mycotic keratitis.

## Methods

### Patient selection and sample collection

Corneal scrapings were undertaken for all patients with suspected infectious keratitis between January 2000 and December 2009. The protocol for the collection of corneal scrapings was approved by the Institutional Review Board at the Vissum Corporación, Instituto Oftalmológico de Alicante, Spain. This research followed the tenets of the Declaration of Helsinki at all times.

Over this decade, 129 cases of keratitis (including bacterial, fungal, viral, Acanthamoeba, and keratitis of unknown etiology) were evaluated in our center. In 20 of them, fungal keratitis was proven by at least one of the three diagnostic tests (stain, culture, or PCR), and the eye condition in all of these patients improved when antifungal drugs were given. Twenty-seven corneal samples were taken of these 20 patients: 21 corneal scrapings, 5 biopsies, and 1 cornea are included in this study. Each corneal sample was processed simultaneously using three methods (smear, culture, and PCR). This was possible as our institute has its own molecular laboratory since 1999.

#### Sample collection and culture for corneal scrapings

Upon completion of the ocular examination and after instillation of topical anesthetic, a sterile Kimura spatula was used to scrape the infected area. Scrapings were inoculated into thioglycolate broth, blood agar, chocolate agar, MacConkey, and Sabouraud's dextrose in aerobic conditions and anaerobic sheep blood agar plate and were placed onto glass slides for staining with Gram and Calcoflour stains. The PCR sample was obtained by scraping and stirring the spatula for a few seconds in 100 μl of sterile water in a 1.5-ml sterile Eppendorf tube. Two aliquots of 50 μl were taken from each sample and stored at −20°C.

#### Sample collection and culture for corneal biopsies

Corneal biopsy was taken in the surgical theater under topical anesthesia for microbiological and molecular analyses. The method involved a double lamellar flap over the lesion. The first lamella was 3 × 3 mm in diameter, over this, and the second lamella was 2 × 2 mm in diameter. A specimen was obtained (1 × 1 mm), from under the corneal flap, in the deep stroma, and then the flap was sutured back into place with nylon 10-0. The surgically excised specimen was trisected for microbiological cultures, stains, and PCR assays. One of the specimens was homogenized and cultured on a solid and liquid phase media, including thioglycolate, blood agar, chocolate agar, MacConkey, and Sabouraud's dextrose in aerobic conditions and anaerobic sheep blood agar plate.

#### Sample collection and culture for cornea

After keratoplasty, the surgically excised cornea was trisected for microbiological cultures, stains, and PCR assays and was processed as in the cornea biopsy cases.

### Smear examination

Glad slides with the corneal specimens were stained with Gram and Calcofluor to be immediately examined under the microscope to detect the presence of any structure compatible with fungus. The Gram-stained slide was examined under optical microscopy for bacteria and fungal pathogens, and the calcofluor-stained slide was examined under fluorescent microscopy light to detect the presence of fungi. All the samples were examined by Gram staining, and all the samples taken after 2005 were examined also with calcofluor staining.

**Fig. 1:**
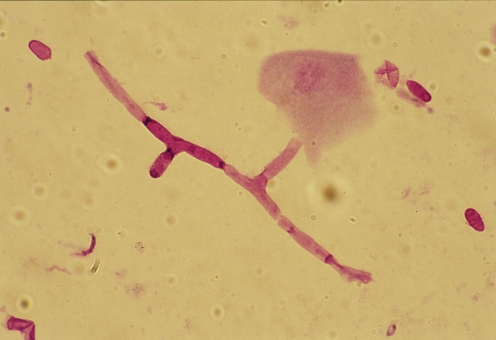
Gram stain examination of corneal scraping of patient 8

**Fig. 2:**
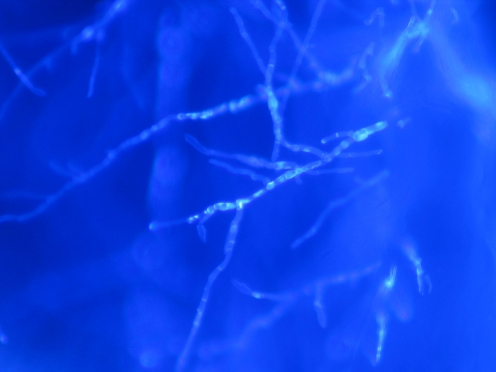
Calcofluor stain examination of biopsy of the patient 19

### Culture

For the microbiological diagnostic test, corneal samples were cultured at 30°C in Sabouraud's dextrose agar and at 37°C in thioglycolate broth, blood agar, chocolate agar, and MacConkey agar. Identification of yeasts was performed by the Auxacolor system (Sanofi Diagnostics Pasteur, Inc, Marnes-la-Coquett, France), and filamentous fungi were differentiated by isolation in Sabouraud chloramphenicol agar and morphological study of macro- and micro-characteristics.

### PCR assay

The DNA extraction and the PCR for fungal DNA detection were performed as described by our group in Ferrer et al. [8].

### DNA sequencing of PCR products

Amplified DNA from PCR was directly sequenced in both directions using the BigDye terminators Ready Reaction Kit (PE Applied Biosystems, Foster City, California) on an ABI Prism automated DNA sequencer (model 377, version 2.1.1; Applied Biosystems Warrington, United Kingdom). The primers used were ITS4 and ITS86.

### Data analysis

DNA sequence was compared to DNA sequences in the BLAST alignment program of the GenBank database (National Institutes of Health) and the EMBL fungal DNA database using Fasta3 sequence similarity searches. The molecular identification was obtained 48 h after the sample was taken.

## Results

Out of the 129 cases with infectious keratitis, 20 cases showed fungal etiology (15.5%). These cases with fungal keratitis were analyzed by stain, culture, and molecular methods. In these 20 cases of keratitis, 21 corneal scrapings, 5 corneal biopsies, and 1 cornea were analyzed. Table 1 shows the results of Calcofluor stain or Gram stain, culture, and PCR of all ocular samples from patients with keratitis of fungal etiology.

**Table 1:** Sample and laboratory diagnosis of fungal keratitis

Patient	Sample	Date	Stain	Culture	Identification culture	Fungal PCR	Molecular identification	Topical/oral treatment
1	Corneal scrape	15/01/2000	Septate hyphae	Positive (2 days)	*Aspergillus fumigatus*	Positive	*Aspergillus fumigatus*	Amphotericin B/fluconazole
2	Corneal scrape	06/10/2000	Negative	Negative		Positive	*Aspergillus niger*	Amphotericin B
3	Corneal scrape	12/02/2001	Negative	Positive (7 days)	*Alternaria alternata*	Positive	*Alternaria alternata*	Amphotericin B/fluconazole
4	Corneal scrape	15/06/2001	Hyphae	Positive (6 days)	*Scedosporium apiospermum*	Positive	*Scedosporium apiospermum*	Natamicin/voriconazole
5	Corneal scrape	19/03/2002	Yeast	Positive (1 day)	*Candida albicans*	Positive	*Candida albicans*	Amphotericin B/fluconazole
6	Corneal scrape	02/08/2002	Hyphae	Positive (7 days)	*Alternaria* sp.	Positive	*Alternaria infectoria*	Amphotericin B/fluconazole
7	Corneal scrape	18/11/2002	Broken hyphae	Negative		Positive	*Aspergillus oryzae*	
8	Corneal scrape	18/02/2003	Septate hyphae and conidia	Positive (1 day)	*Fusarium* sp.	Positive	*Fusarium oxysporum*	Amphotericin B/econazole
9	Corneal biopsy	03/08/2003	Negative	Negative		Positive	*Candida parapsilosis*	
	Corneal biopsy	10/08/2003	Yeast	Positive (2 days)	*Candida parapsilosis*	Positive	*Candida parapsilosis*	Amphotericin B/voriconazole
10	Corneal scrape	07/07/2004	Septate hyphae	Positive (1 day)	*Aspergillus niger*	Positive	*Aspergillus niger*	Amphotericin B
11	Corneal scrape	20/05/2005	Negative	Negative		Positive	*Candida famata*	Amphotericin B/itraconazole
12	Corneal scrape	03/08/2005	Yeast	Negative		Positive	*Candida parapsilosis*	Amphotericin B/voriconazole
13	Corneal scrape	13/10/2005	Negative	Negative		Negative		
	Corneal scrape	20/10/2005	Yeast and hyphae	Negative		Positive	*Candida sake*	Natamicin/itraconzaole
14	Corneal scrape	02/02/2007	Negative	Negative		Negative		
	Corneal scrape	02/02/2007	Negative	Negative		Positive	*Fusarium solani*	
	Corneal biopsy	07/02/2007	Hyphae	Positive (7 days)	*Fusarium solani*	Positive	*Fusarium solani*	Voriconazol/voriconazole
15	Corneal scrape	05/03/2007	Septate hyphae	Negative		Positive	*Pyrenochaeta keratinophila*	
	Corneal biopsy	13/03/2007	Septate and branched hyphae	Positive (6 days)	Unidentified fungi	Positive	*Pyrenochaeta keratinophila*	Natamicin/itraconazole
16	Corneal scrape	07/04/2008	Septate hyphae	Positive (2 days)	Sterile mycelium	Positive	*Paecilomyces* sp.	
	Corneal scrape	03/06/2008	Septate hyphae	Negative		Positive	*Paecilomyces* sp.	
	Cornea	06/06/2008	Septate and branched hyphae	Positive (35 days)	Sterile mycelium	Positive	*Paecilomyces* sp.	Natamicin/itraconazole
17	Corneal scrape	19/08/2008	Negative	Positive (6 days)	Unidentified fungi	Positive	*Scedosporium apiospermum*	Voriconazole/voriconazole
18	Corneal scrape	04/02/2009	Yeast	Positive (1 day)	*Candida* sp.	Positive	*Candida dublinensis*	Fluconazole/fluconazole
19	Corneal biopsy	05/06/2009	Septate and branched hyphae	Positive (2 days)	*Fusarium solani*	Positive	*Fusarium solani*	Natamicin/voriconazole
20	Corneal scrape	23/06/2009	Negative	Positive (2 days)	*Candida* sp.	Positive	*Candida parapsilosis*	Fluconazole/fluconazole

### Efficiency of three diagnosis tests in the case of keratitis

In the first sample taken, 45% of patients with proven fungal keratitis (patients 1, 4, 5, 6, 8, 10, 16, 18, and 19) showed positive results using the three methods (stain, culture, and PCR). Forty-five percent showed positive results by one or two diagnoses methods (patients 2, 3, 7, 9, 11, 12, 15, 17, and 20), and the remaining 10% showed negative results with the three methods (patients 13 and 14).

In the second group, the patients who showed positive results by one or two diagnostic methods were divided into three subgroups: PCR and culture-positive patients (patients 3, 17, and 20), PCR and stain-positive patients (patients 7, 12, 15 and second sample of patient 13), and PCR-positive patients only (patients 2, 9, 11 and second sample of patient 14). Therefore, the PCR was never negative if some of the other two methods were positive. When PCR was positive and the other two methods were negative, another sample was taken depending on the clinical picture. Clinical picture of patients 2 and 11 was typical of fungal keratitis, and these patients did not improve with antibiotic treatment. In these cases, based on the positive result of the PCR, antifungal therapy was initiated, and in both cases, the clinical picture improved. In the cases of patients 9 and 15, a second sample was taken to corroborate the first PCR results due to the fact that they did not show a typical clinical picture of fungal keratitis (patient 9 showed a crystalline keratopathy [9]), or the fungus identified was not a typical fungus found in keratitis (patient 15 showed a keratitis by *Pyrenochaeta keratinophila*, sp nova [10, 11]). In both cases, the second sample results were positive with the three diagnostic methods.

When the results of the three diagnostic methods were negative and the clinical picture deteriorated even when antibiotic therapy was given, a second sample was taken to discard a fungal keratitis (patients 9 and 14). In both cases, the fungal keratitis was confirmed in patient 9 by the three diagnostic methods, and in patient 14, by PCR in the second sample and by the three methods in the third sample taken.

### Efficiency of the three diagnostic tests in the corneal samples

Regarding the efficiency of the diagnostic test in the ocular samples, 66.7% of them were positive by stain, 59.3% by culture, and PCR was positive in 92.6% of samples. The confidence interval (95%) for these proportions was 49% to 84.4% for stain, 40.4% to 78% for culture, and 82.7% to 100% in PCR.

Depending on the type of cornea specimen, stain and culture were positive in 80% of the biopsies and in 61.9% and 52.4% of the corneal scrapings, respectively.

The time taken for PCR assay was 4 to 8 h whereas positive fungal cultures took at least 1 to 35 days.

### Identification of fungal pathogen

DNA database comparison of the DNA sequences obtained with the full-sequence ITS2 and partial-sequence 5.8S rDNA from the ocular samples demonstrated that they were derived from the fungal ITS regions. Thirteen filamentous fungi and seven yeasts were identified. Regarding filamentous fungi, four of them were *Aspergillus* ssp. (two sequences were identical to *Aspergillus niger* ITS2/5.8S rDNA region, and one of each were identical as *Aspergillus fumigatus* and *Aspergillus oryzae* sequences), two of them were *Fusarium* ssp. (*Fusarium oxysporum* and *Fusarium solani*), two of them were *Alternaria* ssp. (*Alternaria alternata* and *Alternaria infectoria*), two of them were *Scedosporium apiospermum*, and one each of *P. keratinophila* and *Paecilomyces* sp. (Table 1).

Regarding yeast, three of them were identical to the *Candida parapsilosis* ITS2/5.8S rDNA region, and one each was identical to the *Candida albicans*, *Candida sake*, *Candida dublinensis*, and *Candida famata* ITS2/5.8S rDNA region (Table 1).

Identification was possible in all cases by molecular methods at species level except in patient 17 (at genus level) and patient 16, where a species nova was discovered, and two identification methods were necessary to describe the species. The identification by conventional methods only was possible at species level in eight of 20 cases.

## Discussion

In this report, we present our experience over the last decade in the detection and identification of fungal pathogens in corneal samples to diagnose fungal keratitis. In this retrospective study, we evaluated the efficacy of the three diagnostic methods, comparing the results of the PCR with stains and culture of 27 corneal samples. As it is a retrospective study, we have been able to evaluate the results of cases with proven fungal keratitis over 10 years, giving us information about when and what type of samples are positive in the progress of fungal keratitis.

The visualization of the corneal scrapings is considered by most clinicians as a rapid and sensitive method in the diagnosis of fungal keratitis. Our study revealed that 66.7% and 59.3% were positive with stain and culture method, respectively. The sensitivity of the visualization of corneal smears as a diagnostic test depends on various factors such as the technique used (KOH, Gram, acrydine orange, calcofluor), the experience of the microbiologist, and the size and sample type. Regarding the technique, we used the Gram and calcofluor stains technique, the former, because it also allows the visualization of bacteria, and the latter, because it is fast and easy to detect fluorescent fungal structures under microscopy (Figs. 1 and 2). With these stains, we have obtained 66.6% of positive results; these data are similar to 61–62% obtained with KOH in other studies [12, 13] or to 60% obtained with Gram by Chowdhary and Singh [13] and much higher sensitivity than 33% [14] or 35% [12] of other studies. Other reasons to explain the diversity in the sensitivity of the corneal sample visualization are the degree of experience of the microbiologist and the size and type of the sample. Our study shows higher sensitivity when a biopsy or cornea is analyzed (83.3%, 5/6 samples) probably due to some fungi that are found in the deep stroma. This highlights the importance of an adequate sample to get a higher probability of positive results; the depth and amount of the corneal sample should be abundant to increase the microbial load.

Finally, one factor which influences the microbiological result is the progression of the keratitis. Our patients are middle-class people and live in urban areas. They come to the center when they feel the first symptom in their eye (early keratitis). However, patients from developing countries, where most of the population live in rural areas, have more difficulty in going to a hospital. Therefore, when they eventually go to a clinic, keratitis is in its advanced stage. This is reflected in the work of Sharma et al. [15]. They studied 477 corneal scrapings from patients with fungal keratitis, of which 114 were patients with early keratitis and 363 were from patients with advanced keratitis. In this study, they proved that fungal detection by calcofluor was much higher in advanced keratitis (sensitivity, 87.1%) than in cases of early keratitis (61%). The last percentage is very similar to the 66.7% we obtained in our study.

Regarding the results of culture, only 59.3% was positive. This sensitivity is higher than in another study (25%) where antifungal therapy was already instituted [12]. This difference between culture and stains may be explained by the fact that the positive result of culture requires viable organisms whereas a stain test can detect both viable and nonviable organisms. In addition, some viable fungal structures in the eye do not grow under laboratory conditions due to the shift of the growth condition (temperature, humidity, and substrate).

In the case of stains, the culture shows higher sensitivity in the corneal biopsies than in scrapings, and the reason is the same.

The combination of microscopy and culture gave positive results in 21 of 27 samples of fungal keratitis (77.7%). PCR detected 25 of 27 samples (92.6%), but if we attend to the cases of keratitis from 20 patients, five of them showed culture and smear stain was negative in the first sample taken; it can also be said that one fourth of the patients with fungal keratitis are not going to have a confirmatory laboratory diagnosis. As the fungal keratitis diagnosis needs to be confirmed by laboratory diagnosis, the patient did not receive antifungal treatment or will have to wait until the second sample is positive for the treatment to be provided.

However, PCR showed 90% of sensitivity if only the samples of the first visit are taking into account. Therefore, PCR is able to reduce the number of patients with fungal keratitis misdiagnosed in the first visit from one quarter to one tenth, preventing patients from being left without treatment or a delay in its application and risking the possibility of losing their vision (and even the eye) that it entails.

In some patients, as in case 16, corneal samples were taken along the infectious process after the application of antifungal drugs. Although the culture was negative after 2 months of treatment, PCR and corneal scraping stain continued showing positive results. Eventually, the patient underwent a cornea transplant because the treatment failed. This shows that although the culture result is negative, if the PCR remains positive, the treatment should not be withdrawn.

Our PCR assay had a sensitivity of 92.6%, which was similar to the sensitivity obtained by Alexandrakis et al. [16] who reported a sensitivity of 89% for their PCR technique used in an experimental model of *Fusarium* keratitis. However, our sensitivity PCR is higher than that reported by Gaudio et al. [14] and Vengayil et al. [12] whose sensitivities of PCR assays in presumed cases of fungal corneal ulcers were reported as 50% and 70%, respectively. The reason for the high sensitivity of PCR shown in our study may be because the selected subjects in our study were all with proven fungal keratitis, in contrast to theirs where the fungal keratitis is presumed. In addition, we used a nested PCR assay that should have shown a higher yield of copy numbers. If we review the bibliography, we can see how the techniques have changed to make it easier, faster, and reproducible for the molecular biologist and useful to clinicians and patients. One of the most important changes is choosing which DNA should be amplified. In the first report of fungal detection in ocular samples, a specific gene (cutinase gene) was amplified to detect DNA of *F. solani*. After this report, all of the works about diagnosis of fungal keratitis are focused on the amplification of rRNA gene region. Ribosomal RNA genes are highly conserved in all fungal species. The use of ribosomal RNA genes for the identification of fungal species is based on the detection of conserved sequences in the rDNA genes. Some authors use the 18S or 28S rRNA gene to detect fungal DNA, but the majority of them (including our group) use as target DNA the region between these two genes, ITSs-5.8S rRNA region (Table 2). The reason for the widespread use of this region is because it allows the design of primers for highly conserved regions (5' and 3' 18S and 28S ends, respectively) but amplifies variable regions that allow us to distinguish between different fungal species (ITS1 and ITS2). Regarding the best technique for identifying the fungal pathogen once the DNA has been detected, it is DNA sequencing because it is able to identify any species of fungi. Some studies show that fungal strains can be distinguished on the basis of size and primary structural differences in the rDNA regions [17–19]. However, although yeast demonstrated a higher level of interspecies variability compared to other fungi, size determination is not precise enough to unmistakably confirm species identification [20]. Other molecular techniques proposed for fungal identification such as the use of hybridization with a specific probe and the specific nested PCR [14, 16] could be useful to confirm a specific fungal infection. However, the broad spectrum of fungi capable of causing keratitis is very high, and if we don’t suspect which fungi it is, it could remain unidentified. The amplification and sequencing of the ITS region eliminate this requirement. In addition, the small size of the fragment permits its sequenciation in both senses (directions) at once, and the obtained sequence provides enough information to identify the fungal species. Regarding the technique used to detect the fungal DNA, the best one to diagnose a fungal keratitis is conventional PCR because it is the only one that allows us to sequence the amplified DNA to identify the fungi. The other techniques (specific nested PCR, DNA microarray, and real-time PCR) are based on specific primers, not on universal primers, and all those keratitis caused by fungi not represented in the pool of specific primers will remain undetected or misidentified. Fungal speciation constitutes an important aid for effective treatment, facilitating the application of species-specific therapy, thereby avoiding problems of drug resistance, and furthermore, establishes a more precise epidemiology. The lack of species identification in corneal infections in the literature prevents precise knowledge of antimicrobial therapy efficiency and species epidemiology. Therefore, whenever possible, we recommend the use of universal or broad-range primers to detect the DNA of the pathogen and its identification by sequencing.

**Table 2:** Fungal DNA detection and identification to diagnose mycotic keratitis

First author	Year	No. of eyes	Target DNA	Detection	Molecular identification	Level of molecular identification
Alexandrakis^16^	1998	3	Cutinase	PCR	Specific PCR	*Fusarium solani*
Ferrer^8^	2001	11	ITSs-5.8S rRNA	Nested PCR	DNA sequencing	Species level
Gaudio^14^	2002	30	18S rRNA	Nested PCR	Nested PCR	*C. albicans, A. fumigatus, F. oxysporum*
Ferrer^21^	2002	1	ITSs-5.8S rRNA	PCR	DNA sequencing	*Alternaria alternata*
Rishi^22^	2003	1	ITSs-5.8S rRNA	PCR	Not done	Not done
Guarro^23^	2003	1	ITSs-5.8S rRNA	Culture	DNA sequencing	*Fusarium polyphialidicum*
Ferrer^24^	2003	1	ITSs-5.8S rRNA	PCR	DNA sequencing	*Alternaria infectoria*
Mancini^25^	2005	1	ITSs-5.8S rRNA	PCR	DNA sequencing	*Scedosporium apiospermum*
Kumar^17^	2005	4	ITSs-5.8S rRNA	PCR	SSCP	Genus level
Kumar^18^	2005	4	ITSs-5.8S rRNA	PCR	SSCP	Genus level
Kumar^19^	2006	4	28S rRNA	PCR	SSCP	Genus level
Ferrer^9^	2006	1	ITSs-5.8S rRNA	PCR	DNA sequencing	*Candida parapsilosis*
Donnio^26^	2006	1	ITSs-5.8S rRNA	PCR	DNA sequencing	*Lasiodiplodia theobromae*
Ghosh^27^	2007	32	ITSs-5.8S rRNA	PCR	DNA sequencing	Species level
Suzuki^28^	2007	1	ITSs-5.8S rRNA	PCR	DNA sequencing	*Malasezia restricta*
Yoon^29^	2008	1	18S rRNA	PCR	DNA sequencing	*Scedosporium apiospermum*
Laich^30^	2008	1	ITSs-5.8S and 28S rRNA	PCR	DNA sequencing	*Alternaria alternata*
Kim^31^	2008	108	18S rRNA	PCR	DNA sequencing	Species level
Embong^32^	2008	30	18S rRNA	PCR	DNA sequencing	Species level
Bagyalakshmi^33^	2008	12	ITSs-5.8S rRNA	PCR	DNA sequencing	*Botryosphaeria Lasiodiplodia* ssp., *Thielavia tortuosa*, *Glomerulla singulata*, *Macrophomina phaseolina*, *Rhizoctonia bataticola*, and *Podospora* spp.
Vengayil^12^	2009	40	28S rRNA	PCR	not done	Not done
Dong^34^	2009	42	ITSs-5.8S rRNA	Microarray	Fluorescent beads	*F. solani, F. moniliforme, F. oxysporum, A. fumigatus, A. flavus*
Badiee^35^	2009	38	18S rRNA	Nested PCR	Nested PCR	*C. albicans*, *A. fumigatus*, *F. solani*
Ferrer^10^, ^11^	2010	1	ITSs-5.8S rRNA	PCR	DNA sequencing	*Pyrenochaeta keratinophila*
Itahashi^36^	2010	40	ITS2	Real-time PCR	Real-time PCR	*Candida* spp.
Menassa 37	2010	15	ITSs-5.8S rRNA	Real-time PCR	Real-time PCR	*C. albicans*, *Aspergillus* spp., *Fusarium* spp.

The second advantage of the PCR technique versus culture is the time to obtain the result; culture took 5.5 ± 8.2 days for a positive growth in our setup (1–35 days). The time to carry out the diagnosis by PCR is about 4–8 h. When PCR was followed by sequencing of the PCR product, the total identification time was 24 h, still significantly faster than culture-based identification. Therefore, PCR-based methods promise to be very effective for the diagnosis of fungal ocular infections in the clinical setting. Compared with standard laboratory techniques, it offers a significant reduction of the time required to establish the diagnosis. PCR may be included in the standard laboratory test together with stain and culture to diagnose fungal keratitis in all the cases where the clinicians suspect fungal keratitis. However, the expertise (skilled hands and high degree of experience) and the cost factor (infrastructure and consumables) may render it in most cases to the smear technique. Therefore, we think that PCR may be performed at least in those cases where the results of corneal scraping stains are negative without waiting for the results of the culture. To establish a protocol to be followed by all laboratories distributed around the world is difficult because the socioeconomic conditions of each country are different. This directly influences the cost–benefit balance, causing it to tilt to either side. A patient with advanced fungal keratitis has a high probability of getting a positive result by staining with calcofluor, although the probability of obtaining a good visual outcome in these cases is also reduced. The cost would be low (smear technique), but the benefit would not be improved by performing a PCR (poor visual prognosis).

However, in patients with early fungal keratitis, the probability of obtaining a positive result by staining is much lower, and the realization of PCR could help to establish an early diagnosis, which is important to get a good visual prognosis. If we wait to obtain the culture result to perform the PCR, the vision of the patient is put at risk. Although more and more laboratories are using molecular biology techniques to support microbiological diagnosis and/or solve some of its limitations, we think it is not sufficient, and PCR may be added for the diagnosis of mycotic keratitis at least in all cases where the result of the corneal scraping stain is negative without waiting for the culture result.
